# Very Early Cognitive Screening and Self-Reported Feeling of Fatigue Three Months After Stroke

**DOI:** 10.3389/fnhum.2021.742105

**Published:** 2021-11-11

**Authors:** Josefin Holmberg, Beatrice Jondell, Tamar Abzhandadze, Katharina S. Sunnerhagen

**Affiliations:** ^1^Department of Rehabilitation Medicine, Sahlgrenska University Hospital, Gothenburg, Sweden; ^2^Department of Occupational Therapy and Physiotherapy, Sahlgrenska University Hospital, Gothenburg, Sweden; ^3^Institute of Neuroscience and Physiology, Rehabilitation Medicine, University of Gothenburg, Gothenburg, Sweden

**Keywords:** stroke, fatigue, cognition, prediction, cerebral infarction, screening, the Montreal Cognitive Assessment (MoCA), self-report data

## Abstract

Stroke is a major cause of disability and the second leading cause of death worldwide. Post-stroke fatigue has been reported as one of the most limiting symptoms after a stroke. Early identification of risk factors for developing post-stroke fatigue is important for providing timely rehabilitation. A correlation has been found between fatigue and cognitive impairment after stroke, but 2 months after stroke at the earliest. In the present study, we examined whether cognitive function screening using the Montreal Cognitive Assessment (MoCA) very early after stroke could explain fatigue 3 months after stroke. A total of 311 stroke patients admitted to a comprehensive stroke unit in Sweden between 2011 and 2016 were included in this longitudinal study. Cognition was screened within 2 days after admission to the stroke unit. Data on self-reported feeling of fatigue were retrieved from Riksstroke’s 3-month follow-up form. The data were analyzed using binary logistic regression. We found that the cognitive function in an acute phase after stroke could not explain self-reported feeling of fatigue in a later stage. The correlation between cognitive impairment and fatigue that has been reported may be detectable no earlier than the subacute phase of stroke. As previous studies have shown that functional outcome, severity of stroke, and sex also correlate with fatigue after stroke, we controlled for these variables in our analysis. In line with previous studies, we found that female patients had higher odds of experiencing fatigue. This is something that health care professionals should be aware of when working with stroke patients.

## Introduction

The global burden of stroke is increasing due to the world’s growing and aging population (Béjot et al., 2016). Stroke is now the second leading cause of death and a major cause of disability worldwide (Katan and Luft, 2018). The advances in stroke treatment have led to an increased proportion of patients who can be discharged to their home from the stroke units (Walsh et al., 2015). As a consequence, symptoms that have previously been overlooked, such as fatigue, have been illuminated as having a major impact on an individual’s subjective quality of life (Graber et al., 2019). Post-stroke fatigue has been defined as a subjective feeling of physical and/or mental exhaustion or a constant lack of energy that has a negative impact on quality of life and causes obstacles in the execution of daily chores (Chen and Marsh, 2018). It has been noted to be one of the most persisting symptoms after stroke (Broussy et al., 2019) and is linked to crucial deterioration in domains of everyday life (e.g., family, social life, and work life) (Graber et al., 2019). The prevalence of fatigue has been reported to be 50% during the first 2 years after stroke (Cumming et al., 2016). Unlike normal tiredness, post-stroke fatigue does not disappear with rest, and, for some patients, the fatigue can become permanent (Chen and Marsh, 2018).

Impaired cognition is another common consequence of stroke (Sexton et al., 2019), but reported prevalence differs based on time for assessment. For example, 74 and 41% of the patients reported post-stroke cognitive impairment in median 2 days and 1 year after stroke, respectively (Horstmann et al., 2014; Sexton et al., 2019). The pooled prevalence of impaired cognition during the first year after stroke was 38% (Sexton et al., 2019). Positive correlation has been reported between impaired cognition and post-stroke fatigue; In these studies, fatigue and cognitive impairment after stroke were assessed between 2 months and 6.1 years after the stroke event (Johansson and Rönnbäck, 2012; Pihlaja et al., 2014; Graber et al., 2019). Impairments in processing speed, working speed, attention, and working memory were significant predictors of fatigue in stroke patients when measured an average 6.1 years after stroke (Johansson and Rönnbäck, 2012). Furthermore, patients who experienced fatigue 3 months after a stroke had significant impairments in processing speed 3 and 6 months after stroke compared to stroke patients who did not experience fatigue at the same time point (Pihlaja et al., 2014). Patients who experienced fatigue have also had impaired memory compared to patients who did not experience fatigue (Pihlaja et al., 2014). The relationship between cognition and fatigue during the acute and early subacute phases of stroke [defined as the first week and 3 months after post-stroke (Bernhardt et al., 2017)] remains unclear.

Functional outcome, severity of stroke, and sex were other factors that correlated with post-stroke fatigue (Christensen et al., 2008; Wang et al., 2014; Chen et al., 2015; Hawkins et al., 2017; Chen and Marsh, 2018). In addition, a higher degree of functional disability was significantly associated with a higher degree of post-stroke fatigue in both the acute phase and subacute phase (i.e., up to 6 months after stroke) (Wang et al., 2014; Chen et al., 2015; Chen and Marsh, 2018). Females were also more likely than males to experience post-stroke fatigue after the subacute phase (Christensen et al., 2008; Chen and Marsh, 2018).

There are many negative effects of post-stroke fatigue and, when it becomes long-lasting, it can hinder return to work or taking part in social activities even though an individual has otherwise recovered (Jonasson et al., 2018). As length of hospital stay after stroke has been decreasing and an increased proportion of patients are discharged to their home, it is important to try to identify early factors explaining post-stroke fatigue. In previous studies on fatigue and cognition, cognitive assessments were made 2 months post-stroke at the earliest and most of the evaluations were performed with neuropsychological batteries. It remains unclear if cognitive function, screened with a short screening tool during the acute phase of stroke, can explain fatigue later after stroke. If it is possible to confirm cognitive function as an early predictor of post-stroke fatigue, rehabilitation could be better planned and adjusted to the limitations of the condition. This is important because many patients feel that their needs are unmet regarding rehabilitation of fatigue (Mckevitt et al., 2011).

The aim of the present study was to investigate whether cognitive function screened 36–48 h after admission to the stroke unit could explain self-reported feeling of fatigue in a later stage after stroke. The available follow-up data were collected 3 months after stroke, which is often the case because patients are normally scheduled for a follow-up appointment at this time.

## Materials and Methods

### Study Sample

This was a longitudinal cohort study. The data were retrieved from a research database in which patients admitted to a comprehensive stroke unit at Sahlgrenska University Hospital (SU), Gothenburg, Sweden, between 2011 and 2016 were registered (Sunnerhagen et al., 2013). The stroke unit has a regional responsibility for thrombectomy. The research database was linked to the Swedish Stroke Register, Riksstroke (Asplund et al., 2011). The Riksstroke is a hospital-based register that covers all Swedish hospitals treating stroke. Data from acute and 3-month follow-up forms were used.

Inclusion criteria were having had a confirmed stroke according to the World Health Organization’s definition (i.e., rapidly developing clinical signs of a focal or global disturbance of cerebral function, symptoms lasting ≥24 h or leading to death, and no apparent cause other than of being vascular in origin), ≥18 years old at the time of the stroke, completed the cognitive screening within 36–48 h after admission to the stroke unit, and completed the Riksstroke’s 3-month follow-up form. The data were not analyzed for patients who answered “don’t know” to the question regarding self-reported feeling of fatigue at 3 months, or were deceased.

### Procedure

For the research database, cognitive function and basic activities of daily living (ADL) were screened by occupational therapists using the Montreal Cognitive Assessment (MoCA) and the Barthel Index (BI), respectively. The screenings were performed 36-48 h after admittance to the stroke unit. During the study period, certification on the MoCA suggested by the copyright holders was not required. However, the occupational therapists had training on MoCA administration according to the hospital’s standards. Stroke-related neurological impairments were assessed by the nurse or doctor in charge when patients arrived at the hospital using the National Institutes of Health Stroke Scale (NIHSS).

The data in the Riksstroke acute form were registered by the heath care staff working in the stroke unit. The 3-month follow-up form was sent by post from the stroke unit to the patients. It was completed either by the patients themselves or, when needed, by a relative or health care staff. If the form was not returned within 1 month, a reminder letter was sent, or a nurse called the patient.

The research database and Riksstroke data were merged by a statistician in Riksstroke using the patients’ personal identification numbers. The received data file did not contain any personal identification number.

### Ethics and Informed Consent

The regional ethical review board in Gothenburg approved the study (042–11, amendment T 966-17). The data file did not contain personal identification number. Informed consent: according to the Swedish Data Protection Authority, the handling of data generated within the framework of quality registers represents an exception from the general rule requiring written informed consent from the patients. Furthermore, the Personal Data Act (Swedish law #1998:204, issued April 29, 1998) allows data from medical charts to be collected for clinical purposes and quality control without written informed consent.

### Outcomes

The outcome variable was self-reported feeling of fatigue 3 months after stroke (Glader et al., 2002). The question was formulated on the Riksstroke’s 3-month follow-up form as follows: “Do you feel tired? If you are tired this question applies regardless of the reason for the tiredness.” The patients had multiple choice answers to choose from: (1) Never or almost never, (2) Sometimes, (3) Often, (4) Constantly, and (5) Don’t know. In this study, the answer alternatives were dichotomized as follows: alternatives 1–2 were coded as not tired (“0”) and alternatives 3–4 were coded as tired (“1”). As noted above, alternative “5” was excluded from the analysis. The dichotomized self-reported feeling of fatigue was used as the dependent variable.

Cognitive function was screened within 36–48 h after admission to the stroke unit. The MoCA is a valid and reliable screening tool for mild to moderate cognitive impairment, with a score range of 0–30; a higher score represents better cognitive function (Nasreddine et al., 2005). The MoCA score was trichotomized; scores ≤19 were coded as “functionally impaired,” 20–25 as “mildly impaired,” and ≥26 as “normal cognitive function” (Toglia et al., 2017). Cognitive function as screened by the MoCA was the primary explanatory variable.

Stroke severity at admission to the hospital was assessed using the NIHSS. The score range was 0–42, with a lower score representing better neurological function (Goldstein et al., 1989). The NIHSS score was dichotomized as follows: ≤2 points indicated mild stroke and ≥3 points indicated moderate to severe stroke.

The patient’s independence in basic ADL was evaluated with the BI within 36–48 h after admission to the stroke unit. The score range was 0–100, with higher scores indicating higher levels of ADL independence (Mahoney and Barthel, 1965). A score ≥95 indicates independence in basic ADL. The variable was dichotomized to “dependent” (≤90 points) and “independent” (≥95 points) (Balu, 2009).

Patients’ characteristics, such as previous stroke (yes/no), age, and sex (male/female) were also analyzed. Age was dichotomized based on the general age of retirement in Sweden, which is 65 years. Functional outcome 3 months after stroke was estimated with the modified Rankin Scale (mRS). The mRS was calculated from Riksstroke’s questions (Eriksson et al., 2007) and dichotomized to functionally independent (mRS 0–2) and functionally dependent (mRS 3–5) (Eriksson et al., 2007).

### Statistical Analysis

The Mann-Whitney *U* test was used for group comparisons regarding continuous variables, and the *z* score was reported when necessary. For the binary and nominal variables, the chi-squared test was used. Subgroup analyses were performed to explore the relationship between cognitive function screened with MoCA and self-reported feeling of fatigue in male and female patients and functionally independent (mRS 0–2 p) and functionally dependent (mRS, 3–5 p) patients. The correlations between self-reported feeling of fatigue, cognitive function, ADL, stroke severity, sex, age, and previous stroke was explored. The correlation between sex and previous stroke was studied using the phi correlation coefficient. Correlations between remaining variables were studied using Spearman’s rank order correlation coefficient. The correlation coefficients were interpreted as small if less than ±0.29 and medium if from ±0.30 to ±0.49 (Cohen, 1992).

Binary logistic regression analyses were performed to identify whether self-reported feeling of fatigue 3 months after stroke can be explained by cognitive function screened with the MoCA 36–48 h after admission to the stroke unit. The dependent variable was self-reported feeling of fatigue (coded as no = 0 and yes = 1). The primary explanatory variable was cognitive function screened by the MoCA. The other explanatory variables were sex (male/female), age (dichotomized using 65-year-old cut-off), stroke severity (NIHSS ≤ 2 p mild stroke, ≥ 3 p moderate to severe stroke), ADL (dependent, BI ≤ 90 p, independent, BI ≥ 95 p), and having a previous history of stroke (no/yes). The variables were selected based on earlier studies and clinical experience (Chen and Marsh, 2018; Paciaroni and Acciarresi, 2019). The analyses were performed as follows:

• The assumptions of the binary logistic regression were checked. All explanatory variables were controlled for a minimum of five observations per dependent variable category.
• The collinearity between the variables was explored, and variables with correlation coefficients less than ±0.7 were included in the same model.
• Three individual models were built for making the results interpretable. Model A was a univariable model that evaluated if very early cognitive function screened by the MoCA can explain self-reported feeling of fatigue 3 months after stroke. In this model we wanted to explore the individual contribution of cognition on self-reported feeling of fatigue. Model B included patient sex and age together with the cognitive function. Model C included ADL, history of a previous stroke, and stroke severity together with cognitive function. Models B and C were multivariable.
• The regression models were evaluated according to the following measures: Hosmer-Lemeshow test (*p* ≥ 0.05 indicated good fit of the model), the area under the curve (AUC > 0.5 indicates that the model is acceptable, though a higher value close to 1 is preferred), and Nagelkerke pseudo R^2^ (value near 1 is best).

The data were analyzed using SPSS Statistics for Windows, version 25.0 (IBM, Armonk, NY, United States). All statistical tests were two-sided with a significance level of 5%.

## Results

### Study Participants

**FIGURE 1:**
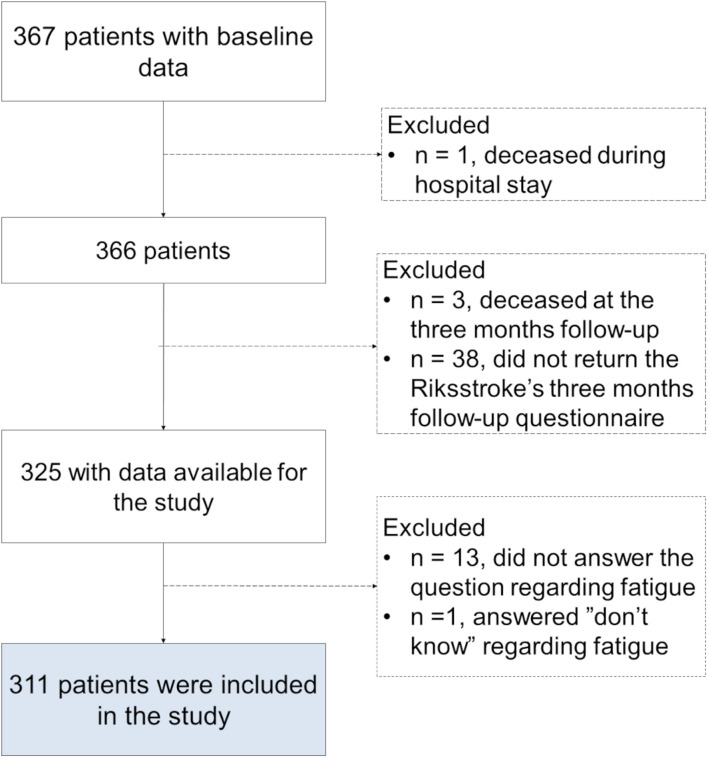
Flowchart of study inclusion.

Of the 367 patients registered in the data file, 311 fulfilled the inclusion criteria and were included in our analysis (Figure 1). There were no significant differences between the included patients (*n* = 311) and excluded patients (*n* = 56) regarding age (*p* = 0.60), sex (*p* = 0.92), or stroke severity (*p* = 0.34).

Of the 311 patients included in the study, 180 (58%) were male, 64.3% had a mild stroke (NIHSS ≤ 2 p) at admission to the hospital, and 51.8% of the patients had impaired cognitive function 36–48 h after admission to the stroke unit (MoCA ≤ 25 p). Other baseline characteristics of the participants are given in Table 1.

**TABLE 1:** Baseline characteristics of the study participants (*n* = 311).

**Characteristic**	**Total sample *n* = 311**
Sex, female, *n* (%)	131 (42)
**Age**	
Age, mean ± SD	69 ± 14.5
Age <65 years *n* (%)	108 (35)
***Accommodation prior to stroke n (%)***	
Own accommodation without home help	285 (92)
Own accommodation with home help	24 (7.7)
Arranged accommodation	2 (0.6)
***Household prior to stroke, n (%)***	
Living alone	131 (42)
Shared household	180 (58)
***Risk factors/comorbidity n (%)***	
Diabetes	39 (13)
Smoker	45 (15)
Previous stroke	54 (17)
Treated for hypertension at onset of stroke	153 (49)
***Reperfusion treatment, thrombolysis, and/or thrombectomy, n (%)***	
Yes	73 (24)
No	229 (76)
***Stroke type and classification, n (%)***	
Ischemic	285 (92)
Total anterior circulation stroke	3 (1)
Partial anterior circulation stroke	48 (15)
Anterior circulation syndrome	102 (33)
Lacunar syndrome	132 (42)
Hemorrhagic	26 (8)
***Stroke-related outcomes, median (range)***	
Cognition, MoCA score	24 (3–30)
Stroke severity, NIHSS score	2 (0–19)
ADL, BI score	95 (10–100)
Length of hospital stay, days	6 (0–37)
***Discharge destination from the stroke unit, n (%)***	
Own accommodation	274 (88)
Arranged accommodation	10 (3)
Another acute clinic	5 (2)
Geriatric/rehab unit	22 (7)

*The sums may vary due to missing data.*

*SD, Standard Deviation; MoCA, Montreal Cognitive Assessment; NIHSS, National Institutes of Health Stroke Scale; ADL, Activities of Daily Living; BI, Barthel Index.*

*The MoCA and BI were administered 36–48 h after admission to the stroke unit.*

*The NIHSS score was determined at admission.*

### Characteristics of the Patients 3 Months Post-stroke

Three months after a stroke, most of the patients (81%) were living in their own accommodation without home help, 62% were living in a shared household and 68% were functionally independent (mRS ≤ 2), Table 2. Regarding self-reported feeling of fatigue, 27 patients (9%) reported feeling fatigued never/almost never, 184 patients (59%) reported feeling fatigued sometimes, 79 patients reported feeling fatigued often (25%), and 21 patients (7%) reported feeling fatigued constantly (Table 2).

**TABLE 2:** Characteristics of the 311 study participants 3 months after a stroke.

**Characteristic**	**Total sample *n* = 311**
***Accommodation, n (%)***	
Own accommodation without home help	252 (81)
Own accommodation with home help	40 (13)
Arranged accommodation	12 (4)
Other (hospitals, rehabilitation units, other)	4 (1)
***Household, n (%)***	
Living alone	114 (37)
Shared household	192 (62)
***Self-reported feeling of fatigue, n (%)***	
Never or almost never	27 (9)
Sometimes	184 (59)
Often	79 (25)
Constantly	21 (7)
***Functional outcome, n (%)***	
Independent (mRS 0–2)	213 (68)
Dependent (mRS 3–5)	92 (30)

*The sums may vary due to missing data.*

*mRS, modified Rankin Scale.*

**FIGURE 2:**
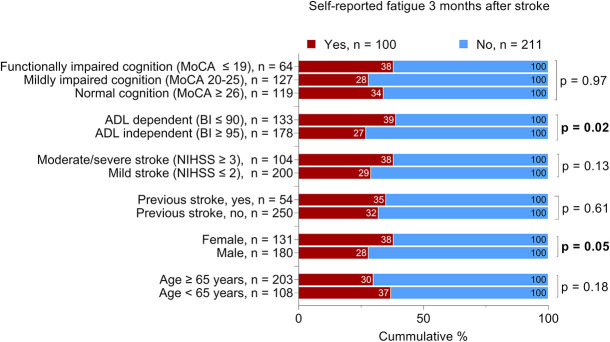
Proportional distribution of the patients with self-reported feeling of fatigue 3 months after stroke. The total number of the patients per variable may vary due to missing data; Bold text indicates statistically significant results. Chi-squared was used for all analyses except for cognitive function, which was analyzed by the Mann-Whitney *U* test. MoCA, Montreal Cognitive Assessment; BI, Barthel Index; NIHSS, National Institutes of Health Stroke Scale. The MoCA and BI were administered 36-48 h after admission to the stroke unit. The NIHSS score was determined at admission.

We found no significant difference between the patients with self-reported feeling of fatigue and patients without self-reported feeling of fatigue regarding the severity of cognitive impairment, stroke severity, history of previous stroke, or age. However, we identified a significant difference regarding sex and ADL ability 36–48 h after admission to the stroke unit (Figure 2).

#### Additional Exploratory Subgroup Analyses

**FIGURE 3:**
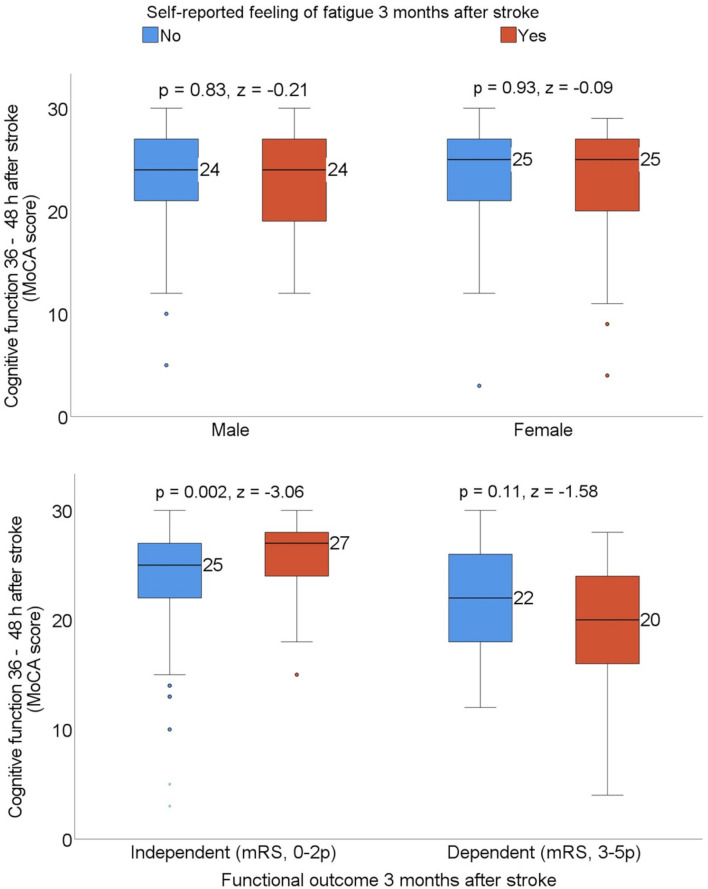
Box plots showing the median Montreal Cognitive Assessment (MoCA) score by sex and patients who were dependent vs. independent 3 months after stroke, stratified with the self-reported feeling of fatigue. Statistics were determined by the Mann-Whitney *U* test. mRS, modified Rankin Scale.

Exploratory subgroup analyses were carried out based on the patients’ sex and functional outcome 3 months after stroke to investigate whether the median MoCA score differed in patients with and without self-reported feeling of fatigue. The median MoCA score 36–48 h after admission to the stroke unit was significantly higher among patients who were experiencing self-reported feeling of fatigue when they also reported functional independence 3 months after stroke (Figure 3).

### Explaining Self-Reported Feeling of Fatigue After Stroke

**FIGURE 4:**
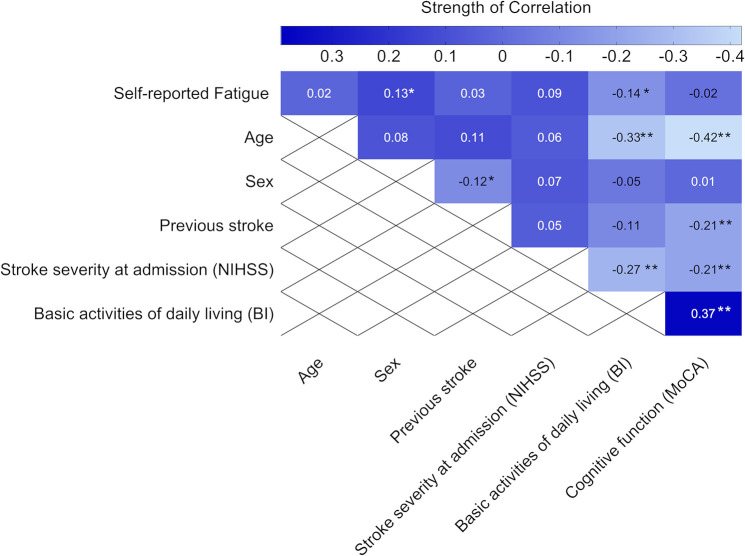
Heat map showing the correlation coefficients (*P*-values) between self-reported feeling of fatigue 3 months after stroke and baseline characteristics (*n* = 311). Statistics were determined by the phi correlation coefficient for binary variables and Spearman correlation coefficient for ordinal variables. Bold text indicates significant results. *p < 0.05 and **p < 0.01. NIHSS, National Institutes of Health Stroke Scale; MoCA, Montreal Cognitive Assessment; BI, Barthel index. The MoCA and BI were obtained 36–48 h after admission to the stroke unit.

Self-reported feeling of fatigue 3 months after stroke was negatively associated with the ability to perform basic ADL, as assessed by BI 36–48 h after admission to the stroke unit. We also found a positive correlation between self-reported feeling of fatigue and female sex, but the strength of the correlation was weak (Figure 4).

The binary logistic regression analyses showed that cognitive function screened with the MoCA 36–48 h after stroke could not explain self-reported feeling of fatigue as an independent variable, or when adjusted for covariates (Table 3). However, female patients had 64% higher odds of self-reported feeling of fatigue compared to male patients (OR 1.64, 95% CI 1.01–2.67, and *p* = 0.05). Age, stroke severity at admission to the hospital, previous history of stroke, and ADL ability 36–48 h after admission to the stroke unit could not explain self-reported feeling of fatigue (Table 3).

**TABLE 3:** Binary logistic regression analyses for explaining self-reported feeling of fatigue 3 months after stroke.

**Model**	**Variable**	**Odds ratio (95% CI)**	***P*-value**	**Nagelkerke R^2^**	**H-L test**	**AUC (95% CI)**
A				0.01	1	0.55 (0.48–0.62)
	Mildly impaired cognition (MoCA 20–25 p)	0.73 (0.43–1.26)	0.26			
	Functionally impaired cognition (MoCA ≤ 19 p)	1.16 (0.62–2.17)	0.65			
B				0.04	0.98	0.61 (0.54–0.67)
	Mildly impaired cognition (MoCA 20–25 p)	0.83 (0.47–1.46)	0.51			
	Functionally impaired cognition (MoCA ≤ 19 p)	1.43 (0.72–2.86)	0.31			
	Female sex	1.64 (1.01–2.67)	**0.05**			
	Age ≥65	0.07 (0.38–1.14)	0.13			
C				0.04	0.50	0.60 (0.54–0.67)
	Mildly impaired cognition (MoCA 20–25 p)	0.65 (0.37–1.15)	0.14			
	Functionally impaired cognition (MoCA ≤ 19 p)	0.88 (0.44–1.78)	0.72			
	Having Moderate to severe stroke (NIHSS ≥ 3 p)	1.24 (0.73–2.11)	0.44			
	ADL dependence (BI ≤ 90 p)	1.66 (0.97–2.84)	0.06			
	Previous stroke, yes	1.17 (0.62–2.23)	0.63			

*Three models are presented. Model A is a univariable model with the primary explanatory variable. B and C are multivariable models and include other explanatory variables besides the cognitive function screened with the MoCA.*

*MoCA, Montreal Cognitive Assessment; BI, Barthel Index; NIHSS, National Institutes of Health Stroke Scale; CI, confidence interval; H-L, Hosmer-Lemeshow; AUC, area under the curve.*

*The BI and MoCA were administered 36–48 h after admission to the stroke unit. The NIHSS score was determined at admission to the hospital. Models B and C were multivariable.*

*The bold text indicates statistically significant results.*

## Discussion

The primary aim of this study was to examine whether cognitive impairment screened with the MoCA 36–48 h after admission to the stroke unit was associated with self-reported feeling of fatigue 3 months after the stroke event. The results showed no significant relationship between cognitive function and self-reported feeling of fatigue. However, a correlation was previously reported between cognitive impairment and post-stroke fatigue assessed in a subacute phase of stroke (Johansson and Rönnbäck, 2012; Pihlaja et al., 2014; Graber et al., 2019). Therefore, our results suggest that early cognitive screening with MoCA may not be useful for explaining self-reported feeling of fatigue at a later stage.

Even after adjusting for sex and age, cognitive function screened with MoCA could not explain self-reported feeling of fatigue. However, we found that female patients were more likely to report self-reported feeling of fatigue 3 months after stroke, which is in line with previous studies (Christensen et al., 2008; Chen and Marsh, 2018). The level of statistical significance for the sex variable was marginal for rejecting the null hypothesis (*p* < 0.05) and the overall explained variance for the model was low at 4%. The higher odds of fatigue after stroke in female patients could perhaps be explained by comorbid conditions, such as depression (Zhang et al., 2021). Furthermore, a subgroup analysis based on functional outcome 3 months after stroke showed that the median score on the MoCA 36–48 h after admission to the stroke unit was significantly higher for the patients who were experiencing self-reported feeling of fatigue when they also reported functional independence 3 months after stroke. It can be assumed that functionally independent patients perform daily chores and other activities to a greater extent than functionally dependent patients. The higher score on the MoCA in this group should be interpreted with the awareness of a ceiling effect (Chan et al., 2014). This means that a normal score on the MoCA (≥26) does not exclude the possibility of cognitive impairment (Chan et al., 2014). Patients with normal cognitive function according to the MoCA may experience undetected cognitive difficulties that, in a later stage, make them more affected by fatigue when going back to their previous life, including work, family life, social life, etc.

A limitation of this study is the method of assessing self-reported feeling of fatigue. Depending on how the questions are formulated regarding the experience of fatigue, the answers and prevalence of fatigue can differ between studies (Johansson and Ronnback, 2014; Graber et al., 2019). The lack of a standardized means of assessing fatigue has been pointed out previously (Duncan et al., 2012; Wright et al., 2017). In this study, we used the Riksstroke’s 3-month follow-up questionnaire, which includes one question regarding self-reported feeling of fatigue. This makes it hard to know which parts of post-stroke fatigue we capture. Post-stroke fatigue is defined as physical and mental exhaustion. By asking, “Are you tired? If you are tired this question applies regardless of the reason for the tiredness,” you assume that the patient is aware of all the symptoms that post-stroke fatigue includes, which may not be the case. It poses the risk of missing patients who, for example, are not aware of the mental part of fatigue. Furthermore, the dichotomization of self-reported feeling of fatigue resulted in a rough division of the answers in our analysis. It is possible to argue that answering, “Occasionally,” when asked, “Are you feeling tired,” represents symptoms of post-stroke fatigue, which would mean that we are missing patients who do experience fatigue. However, it is also possible to argue that “occasionally” qualitatively comprises the normal tiredness that people who have not had a stroke experience. Even though fatigue can be pathological, it is also a normal condition (Johansson and Ronnback, 2014). Therefore, we chose to put this alternative together with the answer, “Never or almost never.”

The primary explanatory variable in this study was cognitive function screened with MoCA. Although MoCA is a valid and reliable instrument for use in patients with stroke, some drawbacks should be mentioned. Namely, not all patients can undergo cognitive screening with MoCA (e.g., patients with aphasia, impaired motor function in dominant upper limb) and it lacks the sensitivity to identify inpatients on specific cognitive domains (Demeyere et al., 2016; Abzhandadze et al., 2021). Another limitation of our study is the characteristics of our sample, as two out of three patients in our sample had a mild stroke and the majority of patients had normal or mildly impaired cognitive function, which probably affected the statistical power. Other factors that could affect self-reported feeling of fatigue are the number and type of medications taken by the patients. These factors could not be addressed in this study due to the unavailability of the data. Therefore, it can be regarded as a limitation of this study.

There were from one (model A) to four (models B and C) statistical tests per regression model in this study. The analyses were not corrected for multiple comparisons. Although there are several solutions addressing this issue, the question is whether a correction should be used at all (Armstrong, 2014), particularly since our study was exploratory rather than confirmatory (Streiner and Norman, 2011). By not correcting for multiple comparisons, the risk was taken to tolerate possible false findings rather than reject potential associations due to Type II error caused by correction (Streiner and Norman, 2011). This decision should be considered when interpreting the study results.

## Conclusion

In conclusion, this study contributes to further understanding the correlation between cognitive impairment and subjective fatigue after stroke. Our results suggest that it is not possible to explain self-reported feeling of fatigue after stroke in a subacute phase by screening cognitive function with the MoCA very early after stroke. Studies have shown a correlation between cognitive impairment and fatigue, but this correlation may be detectable no earlier than the subacute phase of stroke. However, without a standardized means of measuring fatigue, it is difficult to draw any conclusions. However, a risk factor for self-reported feeling of fatigue after stroke that is possible to identify early is female sex. Stroke health care professionals should be aware of this when meeting patients with stroke at follow-up. Our results also show that the prevalence of self-reported feeling of fatigue 3 months after stroke is high, which underlines the importance of routinely assessing fatigue in stroke units. If this can be standardized as routine following a stroke, it could facilitate continued investigation of early risk factors for fatigue.

## Data Availability Statement

The data analyzed in this study is subject to the following licenses/restrictions: according to the Swedish regulations (https://etikprovning.se/for-forskare/ansvar/), the complete dataset cannot be made publicly available for ethical and legal reasons. Researchers can request access to the data by emailing the principal investigator KS, ks.sunnerhagen@neuro.gu.se.

## Ethics Statement

The studies involving human participants were reviewed and approved by the regional Ethical Review Board in Gothenburg approved the study (042–11, amendment T 966-17). Written informed consent for participation was not required for this study in accordance with the national legislation and the institutional requirements.

## Author Contributions

JH and BJ: analysis and interpretation of the data and drafting of the manuscript. TA: acquisition of data, conceptualization of the study, analysis and interpretation of the data, and revising the manuscript for intellectual content. KS: design or conceptualization of the study, interpretation of the data, and revising the manuscript for intellectual content. All authors contributed to the article and approved the submitted version.

## Conflict of Interest

The authors declare that the research was conducted in the absence of any commercial or financial relationships that could be construed as a potential conflict of interest.

## Publisher’s Note

All claims expressed in this article are solely those of the authors and do not necessarily represent those of their affiliated organizations, or those of the publisher, the editors and the reviewers. Any product that may be evaluated in this article, or claim that may be made by its manufacturer, is not guaranteed or endorsed by the publisher.
